# Comparative study of dentin remineralization with Nano-amorphous calcium phosphate-modified bioactive restoratives

**DOI:** 10.1016/j.jobcr.2025.04.009

**Published:** 2025-04-18

**Authors:** Garima Tyagi, Sakshi Jain, Shivani Deshwal, Shubham Singh, Nikita Poonia, Shivangi Sharma

**Affiliations:** a Department of Conservative Dentistry and Endodontics, Kothiwal Dental College and Research Centre, Moradabad, Uttar Pradesh, India, 244001; b Department of Conservative Dentistry and Endodontics, Inderprastha Dental College and Hospital, Sahibabad, Ghaziabad, Uttar Pradesh, India-201010

**Keywords:** Calcium, Phosphate, Dentin, Energy dispersive X-Ray spectrometry, Bioactive, Composites

## Abstract

**Objective:**

Modern caries management emphasizes minimally invasive techniques to promote remineralization through a balanced pH and mineral ion availability. Bioactive restorative materials, including giomer (Beautiful II, SHOFU Dental GmbH, Japan), and ACTIVA BioACTIVE (Pulpdent Corp., Watertown, MA, USA), release fluoride, calcium, and phosphate to enhance dentin remineralization. Nano-amorphous calcium phosphate (NACP) is a highly reactive mineral with proven remineralization capabilities due to its high surface area and solubility. The synergistic effects of NACP and the bioactive materials remain underexplored. This study evaluates the remineralization potential of giomer and ACTIVA BioACTIVE with and without NACP integration using energy-dispersive X-ray spectroscopy (EDX) microanalysis and Knoop hardness number (KHN) assessments.

**Materials and methods:**

Four restorative composite materials: Beautifil II, Beautifil II with NACP, ACTIVA BioACTIVE, and ACTIVA BioACTIVE with NACP, were evaluated on demineralized dentin cavities created in non-carious molars. The mineral content (Ca, P, F) was analyzed via EDX, and the microhardness was measured using KHN. Statistical analyses included analysis of variance (ANOVA) and post hoc Tukey tests.

**Results:**

NACP significantly increased calcium and phosphate deposition, with ACTIVA BioACTIVE with NACP demonstrating the highest remineralization (Ca/P ratio: 2.16). Fluoride intensities were higher in the giomer-based materials, promoting fluorapatite formation. KHN analysis revealed that Beautifil II with NACP exhibited the highest hardness, whereas ACTIVA BioACTIVE with NACP showed reduced hardness despite enhanced mineral content.

**Conclusion:**

The incorporation of NACP into giomer and ACTIVA BioACTIVE facilitated the accumulation of calcium and phosphate ions, leading to enhanced mineralization.

## Graphical abstract

**Figure:**
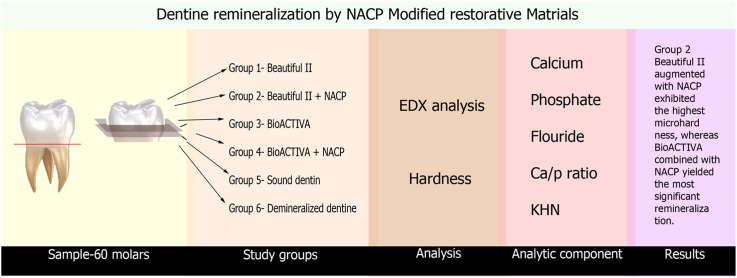

## 1 Introduction

Modern caries management employs a minimally invasive strategy that prioritizes the conservation of partially demineralized dentin, which is regarded as a bioactive matrix conducive to the process of remineralization.^1^ Demineralization can be counteracted when the pH level is balanced and there is an adequate supply of calcium and phosphate ions in the surrounding milieu. This facilitates the reconstruction of partially dissolved apatite crystals, a process known as remineralization. To reinstate natural equilibrium, it is essential to either promote remineralization or inhibit the demineralization process.^2^

Recently, advancements in nanotechnology have facilitated the development of novel resin composites.^3^ These materials have emerged as the preferred direct restorative option in the field of dentistry owing to their capacity to enable minimally invasive or non-invasive procedures, coupled with advantageous characteristics and consistent clinical efficacy. Giomers are formulated utilizing prereacted glass ionomer (PRG) technology. This method involves the pre-reaction of a fluoro-aluminosilicate glass filler with polyacrylic acid, resulting in a stable phase glass ionomer referred to as a “wet siliceous hydrogel.” Subsequently, the fillers were integrated into the resin matrix of the final product. It is posited that the presence of the pre-reacted hydrogel contributes to the fluoride release and recharge characteristics observed in giomer.^4^

ACTIVA BioACTIVE [Pulpdent Corporation, Watertown, MA, USA] is a novel bioactive restorative material introduced in 2013. It combines the physical properties of composites with the ion release characteristics of glass ionomers. This material is capable of releasing calcium, phosphate, and fluoride ions in a sustained manner, which supports dentin remineralization and acid neutralization.^5^ Sajini et al.^6^ conducted an in vitro study in which they evaluated the remineralization potential of giomer and ACTIVA BioACTIVE and concluded that the ACTIVA BioACTIVE showed superior remineralization potential compared to giomer. Nevertheless, the cohort size for the investigation was exceedingly limited (n = 5 for each group), and the authors did not assess fluoride liberation, which constitutes the primary mechanism of action for the remineralization process in giomers.

Nano-amorphous calcium phosphate (NACP) is one of the efficacious methodologies for enhancing dentin remineralization involves the integration of calcium phosphate particulates into adhesive formulations.^7^ Based on this philosophy, NACP has been developed, which is capable of seamlessly flowing with the adhesive into dentinal tubules, thereby facilitating the formation of resin tags.^8^ Notably, NACP has the ability to release calcium and phosphate ions in a "smart" manner, with a heightened discharge of these ions occurring at low cariogenic pH levels.^9^ Furthermore, NACP-infused adhesives can significantly elevate the solution pH from 4 to above 5.5 in a rapid manner, thereby shifting the equilibrium in favor of dentin remineralization.

However, while the use of NACP in dental adhesives is well documented, its incorporation into restorative materials such as giomer or ACTIVA BioACTIVE has not yet been thoroughly investigated. Clarifying this distinction, the current study uniquely focuses on modifying the restorative materials themselves—not adhesives—through NACP addition to evaluate its synergistic effect on remineralization.

This in vitro investigation was initiated to evaluate the dentinal remineralization capabilities of various restorative materials, including Beautifil II restoration (Giomer, SHOFU Dental GmbH, Japan), ACTIVA BioACTIVE, Beautifil II combined with NACP (MilliporeSigma, Darmstadt, Germany), and ACTIVA BioACTIVE integrated with NACP. This was accomplished by assessing the mineral content through energy-dispersive X-ray spectroscopy (EDX) microanalysis and conducting microhardness assessments of the remineralized dentin utilizing the Knoop Hardness Number (KHN). The aim of this study is to investigate whether the incorporation of NACP into bioactive restorative materials enhances their dentin remineralization potential without compromising mechanical performance. The null hypothesis formulated for this investigation asserted that there would be no statistically significant differences between the groups.

## 2 Material and method

### 2.1 Preparation of nanocomposite containing NACP

Commercially available NACP (MilliporeSigma, Darmstadt, Germany) with a particle size of less than 150 nm (20–150 nm) was used. Barium boroaluminosilicate glass particulates with a median diameter of 1.4 μm (Caulk/Dentsply, Milford, DE) were employed as co-fillers. Glass particulates were silanized using a 4 % solution of 3-methacryloxypropyltrimethoxysilane and 2 % n-propylamine (mass %).^10^ A resin formulation comprising bisphenol glycidyl dimethacrylate (Bis-GMA) and triethylene glycol dimethacrylate (TEGDMA) in a 1:1 mass ratio was made light-curable by incorporating 0.2 % camphorquinone (CQ) and 0.8 % ethyl 4-N,N-dimethylaminobenzoate.^8^ This resin was filled with mass fractions of 30 % NACP and 40 % glass particles to form a cohesive paste.

### 2.2 Mixing NACP nanocomposite and beautifil II and ACTIVA BioACTIVE

NACP (40 %) was mixed with 60 % Beautiful II and ACTIVA BioACTIVE on a clean mixing surface, such as a glass slab, using a stainless-steel or glass spatula to achieve a homogeneous paste, avoiding the introduction of air bubbles. The homogenized pastes were preserved under moderate vacuum (2.7 kPa overnight to remove the air that was incorporated during the mixing procedure. The curing systems of ACTIVA BioACTIVE, which uses a dual-cure mechanism (self-cure + light-cure), and NACP nanocomposite using camphorquinone/ethyl 4-N,N-dimethylaminobenzoate are likely compatible because they rely on free-radical polymerization. Beautiful II typically uses CQ-based photoinitiators, similar to the NACP nanocomposite. A small sample of the mixed materials was tested by light-curing them under standard conditions (20 s with a 5-W LED light) to check for proper polymerization (hardened surface, absence of tackiness, and complete depth of cure).

### 2.3 Collection and preparation of samples

**Fig. 1:**
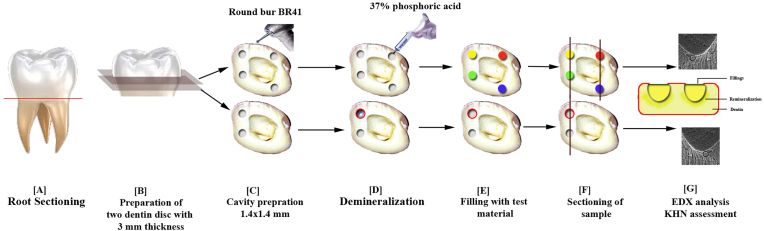
Workflow for the study. A. Sectioning of tooth at cementoenamel junction to separate root, B. Preparation of two dentin discs of 3-mm thickness at middle third of crown, C. Preparation of cavities of size 1.4 × 1.4 mm, D. Demineralization of five cavities except one cavity serving as positive control (marked with red circle), E. Restoration of four test cavities (marked in colors), F. Sectioning of cavities in buccolingual direction to expose junction of base of cavity and remineralized dentin, G. EDX analysis and microhardness testing. (For interpretation of the references to color in this figure legend, the reader is referred to the Web version of this article.)

The sample size for the study was estimated using G∗Power software (version 3.1.9.2 for Macintosh, Heinrich-Heine, Duesseldorf, Germany). One-way analysis of variance (ANOVA) (fixed effects, omnibus) was used to compare the calcium-phosphate (Ca/P) ratio across six groups, including sound dentin and remineralized dentin treated with various restorative materials. Based on an effect size of 0.48 derived from a previous study,^11^ with a significance level (α) of 0.05, and a study power (1-β) of 80 %, the total sample size required was calculated to be 60 samples. This corresponded to 10 samples per group. The research was exempted from ethical approval, given that it constituted an in vitro investigation. The identities of the donors were kept confidential. Non-cariogenic molar teeth were procured for the present investigation from the Department of Oral Surgery. Carious teeth, teeth with cracks, developmental defects, and fillings were excluded. For each specimen, roots were severed to establish a horizontal baseline at cementoenamel junction (Fig. 1A).

A revised iteration of the technique described by Pires et al. was employed to fabricate the specimens.^12^ All teeth were cleaned of soft tissue debris, stains, and calculus, which were then subjected to disinfection using 0.005 % promodyne solution for a duration of 4 h and subsequently preserved at a temperature of 4 °C in distilled water. To prepare dentin specimens in the form of disc, each crown was sectioned in transverse plane at middle 1/3 with thickness of 3 mm for each disc, disc was crafted utilizing a 0.3-mm-thick diamond disc (IsoMet Blade 5LC, Buehler Inc., Lake Bluff, IL, USA) affixed to a precision cutting apparatus (Fig. 1B). Two-disc specimens were obtained from each tooth sample. In the first specimen, four uniformly round dentin cavities, each with a diameter of 1.4 mm and a depth of 1.4 mm, were created using a round diamond bur (BR41, Mani Inc., India) in conjunction with a high-speed dental handpiece equipped with water-cooling features. In the other specimen, two similar cavities were cut to serve as controls (Fig. 1C).

### 2.4 Creation of artificial carious lesions or areas of demineralization

To prevent demineralization of the surrounding tooth structure in disc, an acid-resistant nail polish was uniformly applied to all surfaces except for the cavities.^13^ All cavities except one that served as a positive control were demineralized using 37 % phosphoric acid etchant for 15-s (Fig. 1D). Four cycles at interval of 5 min were repeated for each of the five cavities to create deeper lesions or areas of demineralization, followed by rinsing with water for 60-secs and gentle air drying. Earlier studies conducted demineralization with 37 % phosphoric acid for 60 s.^6^^,^^14^ However, a 60-s exposure to 37 % phosphoric acid represents an extended duration for the process of demineralization, which may result in excessive etching of dentin. Extended exposure to acid can precipitate a significant depletion of calcium and phosphate ions, complicating the assessment of the efficacy of restorative materials in dentin remineralization. Repeated cycles of demineralization used in our study more accurately replicate the frequent acidic assaults that teeth encounter throughout the day as a result of dietary acids, and also ensure the exposure and not destruction of the collagen fibers that are needed for remineralization.

The dentin disc specimen containing two cavities, first specimen with four cavities was subsequently filled with four distinct restorative materials as follows: Group 1 represented a cavity filled with Beautiful II, Group 2 indicated a cavity filled with Beautiful II with NACP, Group 3 denoted a cavity filled with ACTIVA BioACTIVE, Group 4 filled with ACTIVA BioACTIVE with NACP, Group 5 was designated as the positive control, comprising intact dentin devoid of demineralization, while Group 6 served as the negative control, characterized by demineralization absent of any restorative filling (Fig. 1E). All samples were cured as discussed before and then stored in 100 mL artificial saliva (Biotene, GlaxoSmithKline, London, United Kingdom) at 37 °C for four weeks, where the storage solution was changed every two days.

### 2.5 EDX and KHN analysis

After duration of four weeks, the specimens underwent EDX analysis, wherein the samples were sectioned in the buccolingual orientation, precisely along the midpoints of the restorations, utilizing a water-cooled diamond saw to reveal the interface between the restoration and dentin (Fig. 1F). Subsequently, the samples were scanned using EDX (Bruker M4 TORNADO, Bruker Corp., Massachusetts, US). KHN was measured for all samples using a microindentation hardness tester (Zwick Roell ZHU Series, Ulm, Germany) with a 50 g load applied for 15 s at room temperature (23 °C) (Fig. 1G). To ensure accurate results, multiple indentations on different areas of the dentin surface were performed, and the average KHN was taken to account for any surface variation.

### 2.6 Statistical analysis

Statistical analyses were performed using the SPSS software version 23 (SPSS Statistics, Chicago, IL, USA). All data were tested for normality using the Kolmogorov–Smirnov test, and data were found to be normally distributed. One-way analysis of variance (ANOVA) was used to compare the calcium-phosphate (Ca/P) ratio among the six groups. Post-hoc analyses were performed using Tukey's multiple comparison test to identify significant differences between the groups, with a significance threshold set at p < 0.05.

## 3 Results

**Fig. 2:**
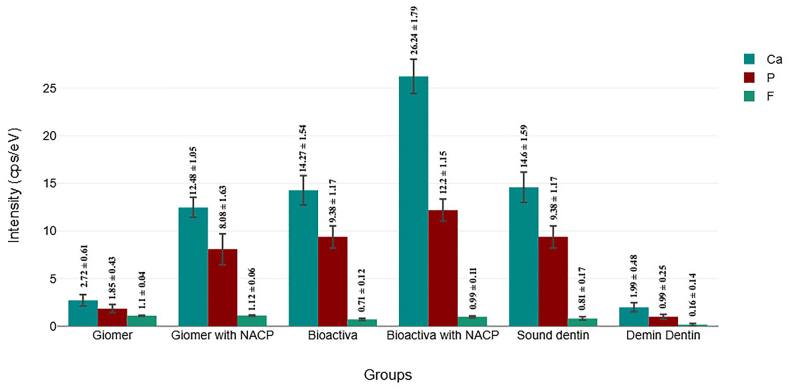
A graphical presentation of EDS analysis showing calcium, phosphate and fluoride intensities (CPS/eV) across different restorative materials.

**Fig. 3:**
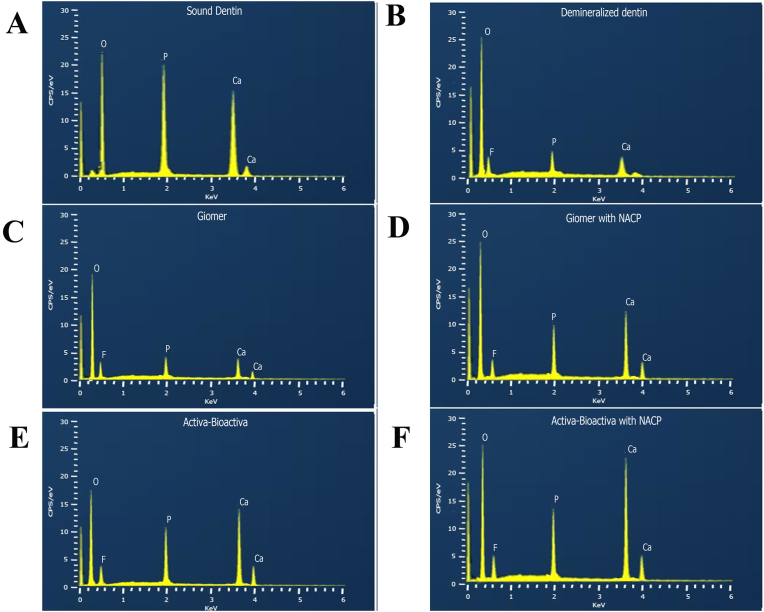
EDS map for [A] Sound dentin, [B] Demineralized dentin, [C] Giomer, [D] Giomer with NACP,[E] ACTIVA BioACTIVE and [F] ACTIVA BioACTIVE with NACP.

The null hypothesis was rejected for the study as EDX analysis (Fig. 2, Fig. 3) revealed significant differences in calcium, phosphate, and fluoride intensities (CPS/eV) among the groups. Beautiful II (Group 1) exhibited relatively low calcium (2.72 ± 0.61 CPS/eV) and phosphate (1.85 ± 0.43 CPS/eV) intensities, while fluoride intensity was higher (1.1 ± 0.04 CPS/eV). In the Beautiful II with NACP (Group 2), calcium (12.48 ± 1.05 CPS/eV) and phosphate (8.08 ± 1.63 CPS/eV) intensities increased significantly compared to the Group 1, while fluoride remained stable (1.12 ± 0.06 CPS/eV). ACTIVA BioACTIVE (Group 3) showed higher calcium (14.27 ± 1.54 CPS/eV) and phosphate (9.38 ± 1.17 CPS/eV) intensities than Group 1, however, the fluoride intensity decreased (0.71 ± 0.12 CPS/eV). ACTIVA BioACTIVE with NACP (Group 4) demonstrated the highest calcium (26.24 ± 1.79 CPS/eV) and phosphate (12.2 ± 1.15 CPS/eV) intensities, while fluoride remained moderate (0.99 ± 0.11 CPS/eV). Sound dentin (Group 5) presented calcium (14.6 ± 1.59 CPS/eV) and phosphate (9.38 ± 1.17 CPS/eV) intensities similar to Group 3, with a slightly higher fluoride intensity (0.81 ± 0.17 CPS/eV). Conversely, the demineralized dentin (Group 6) showed the lowest calcium (1.99 ± 0.48 CPS/eV), phosphate (0.99 ± 0.25 CPS/eV), and fluoride (0.16 ± 0.14 CPS/eV) intensities, reflecting significant mineral loss. These results showed that the addition of NACP significantly enhanced calcium and phosphate deposition, with varying effects on fluoride intensity, contributing to the remineralization potential.

The calcium/phosphate (Ca/P) ratio was also significantly different between the groups (p = 0.001). These results highlight the diverse remineralization capacities of the tested restorative materials. Post hoc analysis further revealed that Group 4 showed a statistically significant difference from Group 5, indicating a significantly higher Ca/P ratio than normal dentin; however, the difference was not significant compared with other groups. Groups 1, 2, and 3 showed statistically significant differences in the Ca/P ratio compared to Groups 4 and 6 (Table 1).

**Table 1:** One way ANOVA for comparison of calcium-phosphate (Ca/P) ratio between groups.

Groups	95 % CI of mean	Mean ± SD	F value	p value	Post hoc Tukey test
**Group 1**	1.36–1.6	1.48 ± 0.16	36.09	0.001∗	Group 1 vs Group 4∗ Group 1 vs Group 6∗ Group 2 vs Group 4∗ Group 2 vs Group 6∗ Group 3 vs Group 4∗ Group 3 vs Group 6∗ Group 4 vs Group 5∗
**Group 2**	1.42–1.75	1.58 ± 0.23			
**Group 3**	1.44–1.62	1.53 ± 0.13			
**Group 4**	2.07–2.25	2.16 ± 0.13			
**Group 5**	1.49–1.63	1.56 ± 0.1			
**Group 6**	1.92–2.11	2.02 ± 0.13			

∗p value < 0.05: Significant, CI: Confidence Interval, SD: Standard Deviation.

The one-way ANOVA demonstrated a significant difference in KHN values across the groups (F = 199.68, p = 0.001). Post-hoc Tukey tests revealed statistically significant differences between most group comparisons (p < 0.05), particularly highlighting Group 4 (53.00 ± 3.53) and Group 6 (27.00 ± 5.52) as having notably lower hardness values compared to other groups. These results indicate substantial variability in dentin hardness, likely attributable to differences in mineral content or structural integrity across groups (Table 2).

**Table 2:** One way ANOVA for comparison of Knoop Hardness (KHN) between groups.

Groups	95 % CI for mean	Mean ± SD	F value	p value	Post-hoc Tukey test
**Group 1**	78.62–82.78	80.70 ± 2.91	199.68	0.001∗	Group 1 vs Group 2∗ Group 1 vs Group 3∗ Group 1 vs Group 4∗ Group 1 vs Group 6∗ Group 2 vs Group 4∗ Group 2 vs Group 6∗ Group 3 vs Group 4∗ Group 3 vs Group 6∗
**Group 2**	80.88–87.52	84.20 ± 4.64			
**Group 3**	69.04–79.16	74.10 ± 7.08			
**Group 4**	50.48–55.52	53.00 ± 3.53			
**Group 5**	75.38–82.62	79.00 ± 5.06			
**Group 6**	23.05–30.95	27.00 ± 5.52			

∗p value < 0.05: Significant, CI: Confidence Interval, SD: Standard Deviation.

The Pearson correlation analysis reveals varying relationships between KHN values and dentin elements across groups. In all groups, KHN values showed a strong positive correlation with calcium (p < 0.001), indicating its critical role in dentin hardness. Positive correlations with phosphate were significant in Groups 3, 4, and 6 (p < 0.05). Conversely, fluoride and Ca/P ratio exhibited inconsistent and weaker correlations, with no significant impact across most groups (p > 0.05), suggesting limited influence on hardness. These findings indicate that calcium and phosphate significantly influenced the KHN of dentin in most groups, while fluoride and the Ca/P ratio exhibited variable correlations depending on the treatment (Table 3).

**Table 3:** Pearson Correlation of Knoop Hardness (KHN) with the elements of dentin.

Groups	Correlation value (r) and p value	Ca	P	F	Ca/P ratio
**Group 1**	r value	−0.28	−0.12	0.96	−0.44
	p value	0.433	0.746	<0.001∗	0.201
**Group 2**	r value	0.97	0.6	−0.55	−0.34
	p value	<0.001∗	0.066	0.096	0.343
**Group 3**	r value	0.92	0.67	0.03	0.22
	p value	<0.001∗	0.036∗	0.938	0.543
**Group 4**	r value	0.96	0.7	0.37	0.02
	p value	<0.001∗	0.024∗	0.287	0.947
**Group 5**	r value	0.95	−0.33	0.26	0.15
	p value	<0.001∗	0.349	0.466	0.686
**Group 6**	r value	0.98	0.93	−0.06	0.07
	p value	<0.001∗	<0.001∗	0.859	0.845

∗p value < 0.05: Significant, Ca: Calcium, P: Phosphorus, F: Fluoride, Ca/P: Calcium-phosphate ratio, Very weak correlation: 0.0<∣r∣<0.20; Weak: 0.2≤∣r∣<0.4; Moderate: 0.4≤∣r∣<0.6; Strong: 0.6≤∣r∣<0.8; Very Strong: 0.8≤∣r∣≤1.

## 4 Discussion

The findings of this study provide a crucial understanding of how the incorporation of NACP into two bioactive restorative materials—giomer and ACTIVA BioACTIVE—influences dentin mineral composition, remineralization capacity, and hardness. The primary objective of this study was to assess the effects of NACP modification on dentin remineralization when added to these established materials. The results revealed notable variances in the intensities of calcium, phosphate, and fluoride, the Ca/P ratio, and hardness among the distinct experimental groups.

### 4.1 Mineral composition and fluoride intensities

Group 1 (Beautiful II) exhibited relatively low calcium and phosphate intensities compared to other groups, reflecting its limited mineralizing potential. Its higher fluoride intensity supports remineralization by promoting the formation of acid-resistant fluorapatite.,^15^ consistent with previous findings.^16^^,^^17^ Fluoride release in giomers is attributed to an ion-exchange process facilitated by PRG fillers, which also enable fluoride recharge when exposed to fluoride-rich environments.^18^, ^19^, ^20^ This rechargeable property helps inhibit acid attacks and supports the repair of demineralized enamel and dentin.

The inclusion of NACP in Group 2 (Beautiful II with NACP) and Group 4 (ACTIVA BioACTIVE with NACP) significantly increased calcium and phosphate intensities while maintaining stable fluoride levels. NACP promotes in situ deposition of calcium and phosphate ions, enhancing enamel and dentin remineralization, aligning with earlier research.^8^^,^^9^ Despite biweekly replacement of artificial saliva, elevated calcium and phosphate levels persisted for 28 days, indicating the sustained remineralization potential of NACP nanocomposites.^21^ These materials also exhibit sustained recharging, allowing prolonged ion release for extended remineralization.^22^

Group 3 (ACTIVA BioACTIVE) demonstrated higher calcium and phosphate intensities than Group 1 but lower fluoride intensity, likely due to differences in fluoride release mechanisms. Unlike giomers, ACTIVA BioACTIVE incorporates bioactive components that facilitate ion exchange and recharge, mimicking natural tooth remineralization processes.^5^ Similar findings have been reported,^6^ though fluoride concentrations were not assessed. ACTIVA BioACTICE's biomimetic properties enable it to promote remineralization through sustained release and recharge of essential ions, replicating the natural replenishment of minerals in healthy teeth.

### 4.2 Ca/P ratio analysis

The Ca/P ratio is vital for assessing dentin's mineral composition, reflecting the balance of calcium and phosphate, key components of hydroxyapatite. Demineralized dentin displayed a high Ca/P ratio (2.02 ± 0.13), likely due to phosphate loss, altering the mineral balance and impairing remineralization. Groups 1, 2, and 3 showed balanced Ca/P ratios, promoting modest remineralization compared to Group 4 with NACP materials. True remineralization restores a natural Ca/P ratio (1.5–1.67). Ratios above 1.67 suggest excess calcium and incomplete hydroxyapatite formation, resulting in weaker, amorphous deposits that fail to mimic natural dentin effectively.^23^ A high Ca/P ratio may result from an excess of calcium relative to phosphate, leading to: Formation of poorly structured or amorphous calcium phosphate deposits, and incomplete hydroxyapatite formation, leaving the repaired dentin weaker and less biomimetic. In such cases, despite the apparent increase in calcium, the remineralization process is inefficient.

### 4.3 Microhardness

Beautiful II with NACP demonstrated the highest hardness, closely followed by Beautiful II, indicating effective remineralization and structural integrity. Groups 1 and 2's high hardness likely stems from fluoride content, promoting acid-resistant fluorapatite formation.^20^ Conversely, ACTIVA BioACTIVE with NACP exhibited reduced hardness, potentially due to altered crystallinity despite high calcium and phosphate levels. Group 4 showed low hardness, reflecting excessive mineralization and a diminished organic matrix, leading to brittleness. The increased Ca/P ratio in Group 4 suggests an imbalance that results in a less resilient and flexible structure, compromising its ability to withstand mechanical stress and mimicking sound dentin inadequately.^24^

### 4.4 Correlation between KHN and dentin elements

Pearson correlation analysis yielded an additional understanding of the association between mineral composition and hardness. In the majority of groups, a positive correlation was observed between KHN and calcium and phosphate levels, especially within groups 1, 2, and 5. This highlights the critical role of calcium and phosphate in preserving dentin hardness. Notably, fluoride did not exhibit a consistent correlation with hardness, indicating that, although fluoride may aid in the process of remineralization, its influence on hardness may be subordinate to that of calcium and phosphate.^25^

One of the key strengths of this study lies in its comprehensive and systematic approach to evaluating dentin remineralization using innovative bioactive restorative materials. By incorporating both NACP-modified and commercially available materials (Beautifil II and ACTIVA BioACTIVE), the study offers a direct comparison of their remineralization potential, enhancing its clinical relevance. The use of human molars, artificial lesion models, and simulation of oral conditions through artificial saliva storage ensures a realistic and biologically relevant setting. Furthermore, the combination of SEM/EDX and microhardness testing provides both qualitative and quantitative assessments, allowing for a more holistic understanding of mineral deposition and structural recovery. The detailed methodology, including standardized cavity preparation and controlled lesion induction, adds to the reproducibility and scientific rigor of the study. Overall, the multi-faceted design and clinically relevant materials strengthen the translational value of the findings, supporting future development and optimization of restorative biomaterials for managing caries-affected dentin.

### 4.5 Clinical implications

This study highlights that adding NACP to giomer and ACTIVA BioACTIVE significantly enhanced calcium and phosphate intensities, promoting dentin remineralization. Giomers with NACP retain higher fluoride levels, making them ideal for caries prevention in pediatric, xerostomic, and orthodontic patients. ACTIVA BioACTIVE with NACP shows superior remineralization potential with the highest Ca/P ratio, which is suitable for restoring severely demineralized areas; however, owing to decreased microhardness, it should be avoided in high-stress areas such as posterior teeth.

### 4.6 Limitations

This in vitro study has several limitations. The controlled laboratory environment does not fully replicate complex oral conditions, such as pH fluctuations, microbial activity, or salivary influence, which can affect remineralization and material performance. The cyclic demineralization process may not accurately simulate the progressive nature of caries in vivo. The long-term effects of ion release and integration into the dentin were not evaluated. Additionally, the study did not assess the influence of mechanical forces such as chewing or brushing on material stability and performance.

## 5 Conclusions

The results of this investigation unequivocally indicated that the incorporation of NACP into giomer and ACTIVA BioACTIVE restoratives facilitated the accumulation of calcium and phosphate ions, leading to enhanced mineralization and an improved Ca/P ratio. Giomers augmented with NACP exhibited the highest microhardness, whereas ACTIVA combined with NACP yielded the most significant remineralization.

## Declaration

During the preparation of this work the author(s) used Paperpal Preflight in order to improve language quality. After using this tool/service, the author(s) reviewed and edited the content as needed and take(s) full responsibility for the content of the publication.

## Authors’ contributions

Garima Tyagi: Conceptualization, Data curation, Formal analysis, Project administration, Methodology, Writing-review and editing.

Sakshi Jain: Data curation, Formal analysis, Methodology, Writing-review and editing. Shivani Deshwal: Conceptualization, Methodology, Writing – review & editing, Project administration.

Shubham: Conceptualization, Project administration, Writing – review & editing.

Nikita Poonia and Shivangi Sharma: Conceptualization, Methodology, Formal analysis, Writing – review & editing.

## Ethical clearance

The present study is a laboratory investigation, which do involve any human tissue (tooth). Ethical clearance was waived off.

## Patient/parent consent

Not applicable.

## Funding

Study was not supported by any internal or external grant or private funding.

## Declaration of competing interest

The authors declare that they have no known competing financial interests or personal relationships that could have appeared to influence the work reported in this paper.
